# Obesity and heart failure with preserved ejection fraction (HFprEF): the whole body question

**DOI:** 10.1016/j.ebiom.2023.104874

**Published:** 2023-11-09

**Authors:** eBioMedicine

Heart failure is a common medical condition in which the heart muscle loses its ability to pump blood efficiently, affecting an estimated 64 million people worldwide. Heart failure has long been a leading cause for hospitalization in people older than 65 in the USA, and the number of people in the USA living with heart failure is expected to rise from an estimated 6.7 million to 8.5 million by 2030 according to the latest statistics released in from the Heart Failure Society of America, which were published in October, 2023.

There are several types of heart failure. The most common forms affect the left chambers of the heart, which specifically work to supply oxygenated blood from the heart to the rest of the body. Newly oxygenated blood that arrives from the lungs is first pumped into the left atrium (top chamber) of the heart, through the left ventricle (bottom chamber), and is then pumped from the heart out to the rest of the body, before returning to complete the cycle by pumping through the right side of the heart, and back to the lungs.

How well the oxygenated blood is pumped out from the left ventricle is measured with a unit known as the ejection fraction (EF), and heart failure sub-types are generally categorized by whether EF is reduced (ie, heart failure with reduced ejection fraction [HFrEF]) or preserved (ie, heart failure with preserved ejection fraction [HFprEF]). HFrEF and HFprEF have distinct causes; HFrEF is associated with cell death and a loss of cardiomyocytes, whereas HFprEF has been associated several chronic and multi-system comorbidities such as obesity, type 2 diabetes, kidney disease, and high blood-pressure. Similar to these chronic conditions, HFprEF is also increasing in incidence. Although several effective treatments are available for HFrEF, effective treatments for HFprEF have lagged, perhaps due to the complex and potentially multi-system nature of its pathophysiological origins.

Given the interdependence among biological systems, clinical trialist think tanks such as MOSAIC (Metabolic and multi Organ Science Accelerating Innovation in Clinical Trials) are integrating cross-disciplinary approaches to trial design. With the end goal of reducing the number of patients who progress to life-threatening cardiovascular disease (CVD), on October 9th, 2023. The American Heart Association issued a presidential advisory regarding a newly designated cardiovascular-kidney-metabolic (CKM) syndrome, urging for a re-evaluation of risk, prevention, and holistic management of the patient in light of system and organ inter-dependencies.

Considering this interconnectedness, some of the most exciting therapies currently being explored for heart failure are two classes of glucose-lowering drugs that were first used to treat people with type 2 diabetes: Sodium-glucose transport protein-2 inhibitors (SGLT2i) and glucagon-like peptide-1 (GLP-1) agonists.

SGLT2 inhibitors, also known as gliflozins or flozins, are thought to reduce blood sugar by inhibiting SGLT2-mediated transport in the kidney—effectively reducing sodium and glucose resorption by the kidney, and leading to increased glucose excretion in the urine. In 2015, the EMPA-REG OUTCOME study, a randomized controlled trial published in the *New England Journal of Medicine*, showed that people with type 2 diabetes who were at risk of cardiovascular disease and given empagliflozin, an SGLT2 inhibitor, in addition to standard care had a 38% relative risk reduction in death from cardiovascular disease and 35% relative risk reduction in hospitalisation for heart failure, after 3.1 years follow-up. Since the publication of this study, SGLT2 inhibitors have been trialled in people with and without diabetes, and have consistently been shown to reduce the risk of hospitalization for heart failure and death from cardiovascular disease. A meta-analysis (n = 21,947) published in *The Lancet* on September 3, 2022, provided support for the use of this class of drug to reduce the risk of negative cardiovascular outcomes across heart failure subtypes. Importantly—people with preserved ejection fraction and mildly reduced ejection fraction benefitted substantially from the use of these drugs, providing a much-needed treatment approach for this group of people.

GLP-1 receptor agonists, including semaglutide, are also promising in the treatment of HFprEF. These drugs reduce blood glucose by stimulating the release of insulin from the pancreas. In addition to widespread use in the treatment of diabetes, GLP-1 receptor agonists have gained attention due to off-label use for the treatment of obesity. In a randomised controlled trial (n = 529) published in the New England Journal of Medicine on September 21st, 2023, people with HFprEF and BMI higher than 30 kg/m^2^, without diabetes, were given semaglutide or placebo and followed up for 1 year. Patients not only lost weight, but had improved symptom burden and functional impairment metrics associated with HFprEF.

At *eBioMedicine*, we have a longstanding interest in promoting a better understanding of pathophysiological mechanisms. These successes in treatment approaches for HFprEF raise some interesting considerations. The association between obesity and HFprEF seems to be a particularly exciting area of development. For example, research is needed to better understand whether the well-known effects of adipose tissue on metabolic and inflammatory profiles have direct influences on the heart. Clarification of the cellular and molecular pathways that connect these inter-dependent systems with cardiovascular disease outcomes will also be of great interest. Considering the American Heart Association advisory on cardiovascular-kidney-metabolic syndrome, the kidneys should also be included when researching mechanistic connections to improve heart-failure therapies. We welcome studies in this emerging area of holistic research.

